# Graphene and 2D Hexagonal Boron Nitride Heterostructure for Thermal Management in Actively Tunable Manner

**DOI:** 10.3390/nano12224057

**Published:** 2022-11-17

**Authors:** Huibin Sun, Yunlei Jiang, Renjie Hua, Runhua Huang, Lei Shi, Yuan Dong, Suxia Liang, Jing Ni, Chi Zhang, Ruoyu Dong, Yingru Song

**Affiliations:** 1 School of Mechanical Engineering, Hangzhou Dianzi University, Hangzhou 310018, China; 2 Climate School, Columbia University, New York, NY 10027, USA; 3 Zhejiang Business College, BinJiang Campus, Hangzhou 310051, China; 4 Hangzhou Zhongneng Photoeletricity Technology Co., Ltd., Hangzhou 310018, China; 5 School of Physics and Optoelectronic Engineering, Hangzhou Institute for Advanced Study, University of Chinese Academy of Sciences, Hangzhou 310024, China; 6 School of Astronautics, Beihang University, Beijing 102206, China; 7 Department of Mechanical Engineering, William Marsh Rice University, Houston, TX 77005, USA

**Keywords:** thermal management, graphene, hexagonal boron nitride, NEMD, heterostructure

## Abstract

Thermal management is a critical task for highly integrated or high-power semiconductor devices. Low dimensional materials including graphene and single-layer hexagonal boron nitride (BN) are attractive candidates for this task because of their high thermal conductivity, semi-conductivity and other excellent physical properties. The similarities in crystal structure and chemistry between graphene and boron nitride provide the possibility of constructing graphene/BN heterostructures bearing unique functions. In this paper, we investigated the interfacial thermal transport properties of graphene/BN nanosheets via non-equilibrium molecular dynamics (NEMD) simulations. We observed a significant thermal rectification behavior of these graphene/BN nanosheets, and the rectification ratio increased with the system length increases up to 117%. This phenomenon is attributed to the mismatch of out-of-plane phonon vibration modes in two directions at the interface. In addition, we explored the underlying mechanism of the length dependence of the thermal transport properties. The results show promise for the thermal management of this two-dimensional heterostructure in an actively tunable manner.

## 1. Introduction

In the past decade, thermal diodes have been widely studied in many fields, including new energy [1], sensors [2], and especially, integrated circuits (ICs) thermal management [3]. As one of the key factors in determining the performance of the thermal diode, the thermal rectification (TR) effect has drawn much attention [4,5,6]. So far, many methods have been devoted to constructing the TR effect [7,8,9,10,11]. A straightforward way is to combine two materials with different thermal conductivities. Recently, the size of electronic devices has been further reduced, even down to the nanometer scale, which is essential to study the TR phenomenon of nanomaterials [12].

Considerable works have been carried out around one-dimensional, two-dimensional and hybrid one- and two-dimensional nanomaterials [13,14,15,16,17]. Considering the cost and the special crystals structure, two-dimensional graphene heterostructures show great potential for constructing thermal rectification, and researchers have carried out a large amount of work in exploring different heterostructures [18,19,20]. For example, Vallabhaneni et al. [18] studied the thermal transport properties of the graphene/CNT-silicon asymmetric structure interface and calculated a TR of about 20%. Zhang et al. [21] used non-equilibrium molecular dynamics (NEMD) to investigate the interfacial thermal conductance and thermal rectification of graphene/BCnN. They observed that the rectification ratios of BCN/graphene and BC_2_N/graphene were 21% and 13%, respectively. Another piece of work on the thermal energy transfer at the graphene/C_3_N interface using reverse nonequilibrium molecular dynamics (RNEMD) was carried out by Song et al. [19]. They investigated the effects of temperature, nitrogen doping, strain and substrate material on the thermal conductivity of the interface and found the presence of thermal rectification. However, the rectification effect of these heterogeneous structures is very small.

Boron nitride crystals belong to the hexagonal crystal system and are very similar to graphene in structure. At this point, graphene/BN heterostructures are considered to be a potential candidate for the thermal diode [22]. Zhuang et al. [23] obtained TR up to 334% at a temperature difference of 240 K and interfacial chirality angle of 30°. They observed that the TR of the 10.6 nm system was always greater than that of the 21.4 nm length system. Chen et al. [24] investigated the thermal rectification and negative differential thermal resistance behavior of graphene/BN nanosheets. They demonstrated that the optimum conditions for TR include low temperature, large temperature bias, short sample length and small interface densities. As discussed above, some progress has been made; however, studies on the length dependence of the thermal rectification ratio of monolayer graphene/BN heterostructures are still in their infancy.

In this work, we proposed to design an efficient thermal diode based on 2D graphene/BN. We used NEMD to investigate the interfacial heat transport properties of 15–150 nm graphene/BN nanosheets based on the ReaxFF potential. Our group has been working on molecular dynamics simulations based on the ReaxFF potential [25,26,27,28,29,30]. More importantly, our previous work demonstrated the advantages of the ReaxFF potential in calculating the thermal conduction of low-dimensional materials [25]. The results show the presence of thermal rectification, and the rectification ratio increases with length increases. Then, the origin of thermal rectification is analyzed in detail via photon spectra. In addition, we study the length dependence of the thermal rectification ratio from the perspectives of vibration modes. Our work will contribute to the thermal management of ICs.

## 2. Materials and Methods

### 2.1. Simulation Details and Model Setup

**Figure 1:**
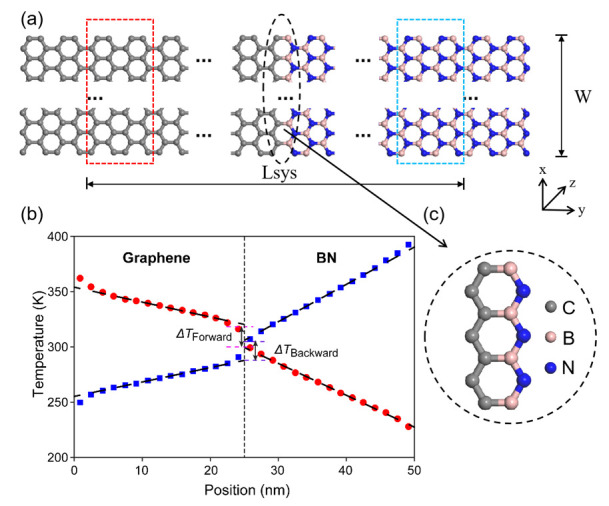
(**a**) Simulation system of the nanosheet thermal diode. It is formed by connecting the graphene and boron nitride (BN) in equal proportion. Red and blue dashed lines represent heating area and cooling area, respectively. Heat flows from graphene to BN (forward) or from BN to graphene (backward). (**b**) Temperature profile of a 50 nm long system reaching a steady state after heat equilibration. The red data points represent the forwards direction, while the blue ones represent the backwards direction. (**c**) Detailed view of the interface formed by C-B-N bonds.

The simulation models, which consist of graphene and boron nitride (BN), are constructed using similar methods described in our previous work [29]. The edge structure of the nanosheet is zigzag in the length direction, as shown in Figure 1a. In our study, the bond length is set to 0.142 nm according to the experimental data [31,32]. The width (*x*-axis) of each system is 4.919 nm and the length (*y*-axis) varies from 15 nm to 100 nm. The first and last unit of atoms in the y-direction are fixed to avoid the spurious global rotation. Next to the fixed atoms, two units of atoms serve as the heating and cooling areas. The periodic boundary conditions are applied in x- and z-directions, while the fixed boundary conditions are applied in the y-direction. To eliminate the effect of periodic action on the simulation results, empty space (size of three units of atoms) is left in both x- and z-directions.

### 2.2. Calculation of Interfacial Thermal Conduction

All NEMD simulations are performed by the LAMMPS package [33]. The ReaxFF forcefield is employed to describe the interaction between C-B-N atoms. In all the simulations, the time step is set to 0.2 fs. The following procedures are included in our simulation runs. First, the graphene/BN nanosheet is relaxed in an NVT ensemble at 300 K for 1 ns. Then, the relaxed system is converted to an NVE ensemble at 300 K for 1 ns for sufficient equilibration. Finally, the heat flux Q, which is set as 1.0841 eV/ps, is applied to the system, according to the method of Ikeshoji and Hafskjold [34]. At each time step, the Q amount of energy is subtracted from the cooling area, while it is added to the heating area. The heat flow from graphene to BN is defined as forward, and the opposite direction is defined as backward. In order to obtain the temperature gradient of the graphene/BN structure, we divide the system into 30 segments along the length direction (*y*-axis). The temperature of each segment is calculated by averaging the kinetic energies of the atoms in each segment:

(1) $$T_{j}=\frac{1}{3k_{B}N_{j}}\sum_{i\in S_{j}}m_{i}v_{i}^{2}$$

where *T_j_* is the local temperature of segment *j*, *N_j_* is the number of atoms in the segment, *m_i_* is the atomic mass, *V_i_* is the atomic velocity, and *k_B_* is the Boltzmann constant. We place the system in a heat bath at 300 K until the average temperature difference ΔT at the interface fluctuates less than 5%, which means the system reaches a stable state. The interface temperature difference is defined as the graphene interface temperature (fitted from the 5th to 14th segments) minus the BN interface temperature (fitted from the 17th to 26th segments), as shown in Figure 1b. We find that the system reaches a stable state after 3 ns, and an additional 2 ns of simulation is conducted to collect the temperature data.

The interfacial thermal conductance, *K*, across the graphene/BN is defined by: K=Q/AΔT, where A = W * δ. Here, *A* is the cross-section area of graphene/BN, ΔT is the temperature drop at the interface, *W* is the width of the system and *δ* is the thickness of the graphene, which is 0.335 nm. The thermal rectification ratio, *R*, is defined as $R=K_{Backward}/K_{Forward}-1$.

### 2.3. Study of Thermal Rectification Mechanisms and Phonon Properties

We calculate the phonon density of states (PDOS) for different systems to probe the mechanism of the thermal rectification effect at the graphene/BN interface. A unit of atoms at the interface (half in the C/BN region) is used to calculate PDOS. The PDOS are extracted from the discrete Fourier transform of the velocity autocorrelation function:

(2) $$D(w)=\int_{0}^{\tau }v(0)\cdot v(t)\exp(-iwt)dt$$

where D(w) is the PDOS at the frequency w. v(0)⋅v(t) is the autocorrelation function of atomic velocities. The velocities are correlated every 0.2 fs over a total integrate time of 0.02 ns after a 5 ns heat bath.

To quantify the degree of overlap of the PDOS, we calculate the cumulative correlation factors (CCF) to describe the matching properties of the phonon modes of atoms at the interface. The CCFs were used by Dong et al. [28] and Diao et al. [26] to quantify the mismatch degree of PDOS. In our work, the CCF is defined by:

(3) $$M_{i}{}_{j}(w_{s})=\frac{\int_{0}^{w_{s}}D_{i}(w)\cdot D_{j}(w)dw}{\int_{0}^{\infty }D_{i}(w)dw\cdot \int_{0}^{\infty }D_{j}(w)dw}$$

where i and j represent individual groups of atoms, and $M_{i}{}_{j}(w_{s})$ is the CCF of PDOS below a specific frequency, $w_{s}$, between i and j.

## 3. Results and Discussion

First, we calculate the thermal conductance K of the 75 nm system to verify the reliability of our approach. The results show that K(forward) and K(backward) equal 4554 MWm^−2^K^−1^ and 5547 MWm^−2^K^−1^, respectively, which is similar to those calculated by others, as summarized in Table 1. It should be noted that the difference between the K(forward) and K(backward) demonstrates the existence of the thermal rectification, which agrees with the results reported by Chen et al. [24] and Zhuang et al. [23]. Then, we analyze the mechanism of thermal rectification in detail in Section 3.1. In addition, we systematically study the several systems with different lengths including 15, 25, 50, 75, 100, and 150 nm to explore the relationship between system length and the thermal properties of graphene/BN in Section 3.2.

### 3.1. Analysis of the Thermal Rectification Mechanism

**Figure 2:**
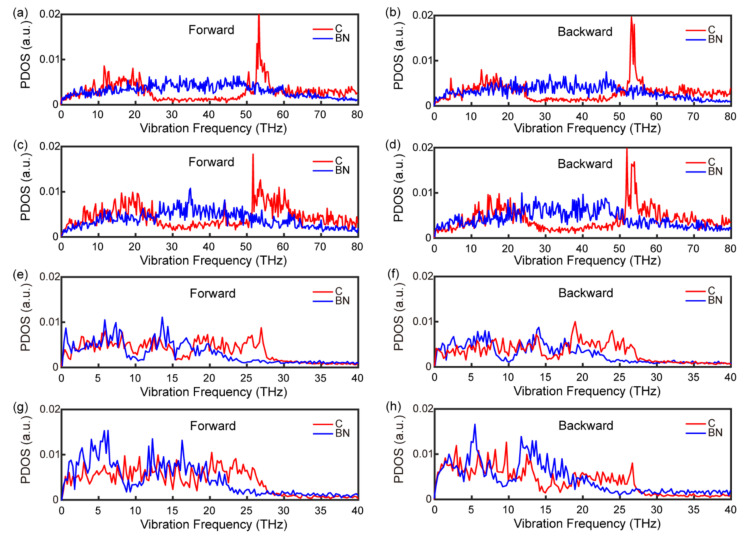
The PDOS curves of carbon (C) and boron nitride (BN) atoms in the forwards and backwards directions. (**a**,**b**) in-plane direction in the middle (a unit of atoms in the middle region of pure C/BN); (**c**,**d**) in-plane direction at the interface; (**e**,**f**) out-of-plane direction in the middle; (**g**,**h**) out-of-plane direction at the interface.

The phonon density of states (PDOS) is a powerful tool for describing phonon information in materials. It has been used in many studies to deeply trace the phonon activity, and thus, to understand the thermal transport properties of materials [37,38,39,40,41]. In order to investigate the origin of thermal rectification, we calculate and plot the PDOS curves for the 75 nm system (Figure 2).

**Figure 3:**
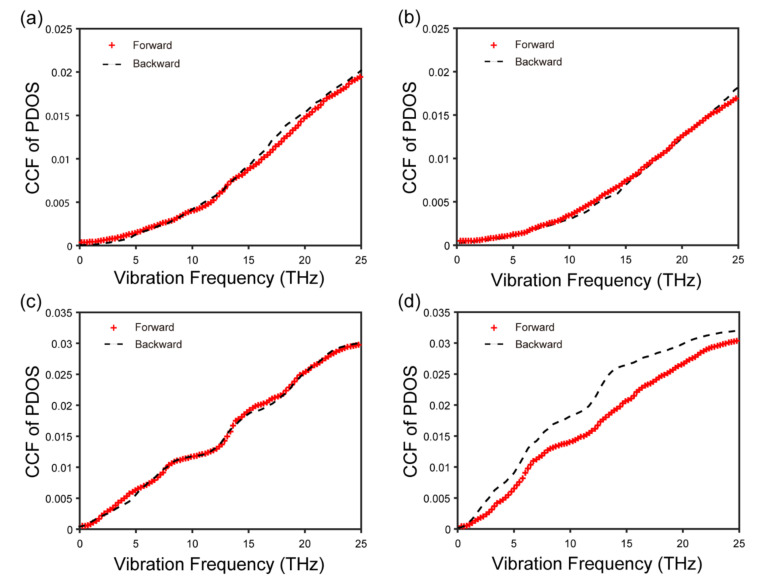
CCF of PDOS between C and BN atoms in the forward and backward direction. (**a**) In-plane direction in the middle; (**b**) in-plane direction at the interface; (**c**) out-of-plane direction in the middle; (**d**) out-of-plane direction at the interface.

We can see from Figure 2a–d that for the in-plane direction, the vibrational modes of both C and BN atoms in the forward direction are similar to those in the backward direction, regardless of the position of the atoms. This verifies that there is no explicit connection between in-plane phonon modes and thermal rectification. In contrast to what is expressed in the out-of-plane direction in the middle (Figure 2e,f), as shown in Figure 2g,h, there is a remarkable difference between these modes of vibration in the out-of-plane direction at the interface, which could be responsible for the existence of the thermal rectification effect. Although, in the forward and backward direction, low-frequency phonons are both the main carriers of heat, the PDOS of C and BN atoms matches better in the backward direction (Figure 2h) than in the forward direction (Figure 2g) in the relative low frequency (≤10 THz), indicating better heat transport in the backward direction [24]. In other words, the heat flux flows preferentially from BN to graphene. In order to quantify the matching degree difference, we further calculate the CCF, which is usually used to reflect the matching degree of the atoms at the interface for PDOS in one heat flow direction [26,28]. As shown in Figure 3a,b, the in-plane CCF curves almost overlap, while the CCF in the backward direction is larger than that in the forward direction (Figure 3d). In addition, the out-of-plane CCF curves in the middle region are well matched (Figure 3c). Therefore, we conclude that the phenomenon of thermal rectification in graphene/boron nitride could arise from the mismatch of out-of-plane phonon vibration modes in two directions at the interface. Meanwhile, the thermal rectification direction (from the BN to graphene) is due to the larger CCF in the backward direction. This is similar to what has been revealed in other graphene heterostructures, such as graphene/C3N [19] and graphene/MoS2 [20].

### 3.2. Length Dependence of Thermal Rectification Ratio

**Figure 4:**
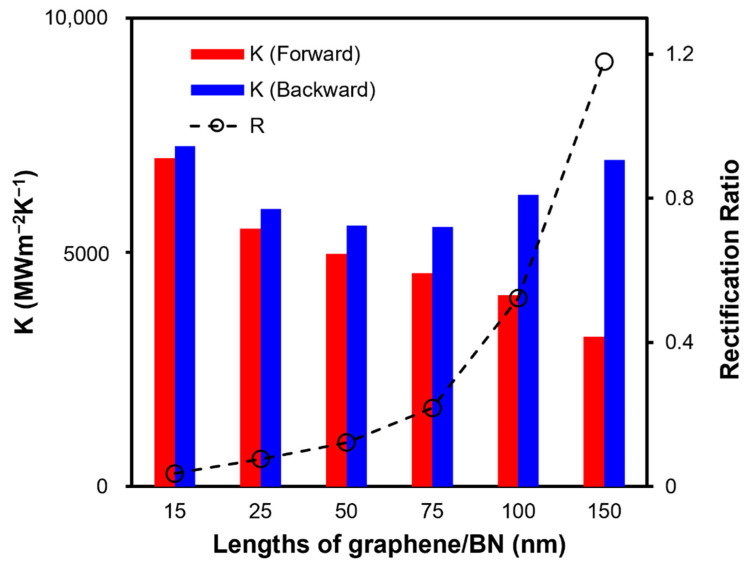
Forward and backward interfacial thermal conductance of graphene/BN with lengths of 15, 25, 50, 75, 100, 150 nm and the corresponding rectification ratio.

Although Chen et al. [24] and Zhuang et al. [23] both observed the TR effect in the graphene/BN system, their conclusions about length dependence are different, and the underlying mechanism of the length dependence remains unexplored. Here, we calculate the interfacial thermal conductance and thermal rectification ratio of graphene/boron nitride nanosheets with different lengths. As shown in Figure 4, the K(backward) is always larger than K(forward). Specifically, in the forward direction, K decreases from 7008 MW m^−2^K^−1^ to 3199 MW m^−2^K^−1^, while in the backward direction, K first gradually decreases from 7263 MW m^−2^K^−1^ to 5547 MW m^−2^K^−1^ and then increases to 6972 MW m^−2^K^−1^. These changes lead to the increase in R from 3% to 117% as the system length increases from 15 nm to 150 nm, which is in contrast to that found by Chen et al. [24] that the R decreases with the increase in system length.

**Figure 5:**
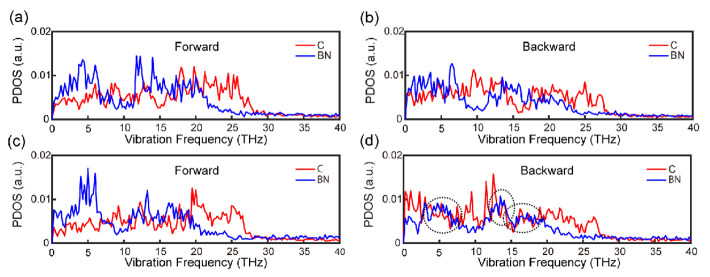
The out-of-plane PDOS curves of carbon and BN atoms at the interface (forward and backward). (**a**,**b**) Graphene/BN-25 nm; (**c**,**d**) graphene/BN-100 nm.

Next, we investigated the relationship between the thermal rectification ratio and length from the perspective of phonon vibration modes. Taking systems of 25 nm and 100 nm in length as examples, we perform a phonon power spectrum analysis and plot the PDOS curves of out-of-plane phonons in Figure 5. In the forward direction, it can be seen from Figure 5a,c that in the frequency range less than 20 THz, the PDOS peaks of BN become higher and narrower when the size of the system changes from 20 nm to 100 nm. Such a change broadens the difference in phonon vibration modes at the interface, which accounts for the decrease in K in the forward direction. Meanwhile, in the backward direction, we can apparently observe that there are three overlapping regions in Figure 5d, indicating a better match between the C and BN atoms. Further, it has been reported that a better match leads to a more profound phonon resonance, directly contributing to higher heat transport [42].

**Figure 6:**
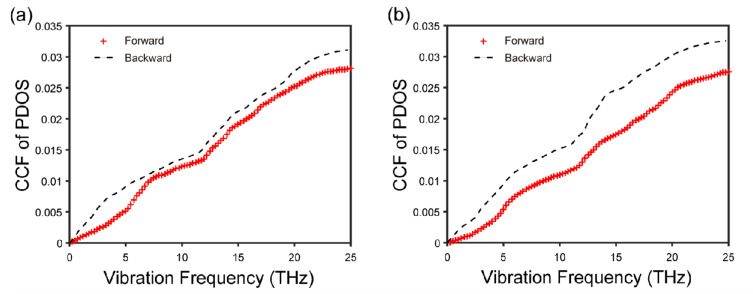
CCF of PDOS between C and BN atoms in the forward and backward direction. (**a**) Out-of-plane 25-nm-graphene/BN; (**b**) out-of-plane 100-nm-graphene/BN.

Then, we further calculate the CCF of PDOS between C and BN atoms in the for-ward and backward direction in frequency ≤ 25 THz. Compared with the system length of 25 nm, the system with a length of 100 nm has a larger matching degree difference (Figure 6). This explains why R increases from 3% to 117%. In addition, this implies that we can obtain very high thermal rectification ratios by increasing the length of the system, which is important for the design of thermal diodes.

## 4. Conclusions

In summary, we used non-equilibrium molecular dynamics simulations to investigate the interfacial thermal transport properties of graphene/BN nanosheets based on ReaxFF potential. We found that the thermal rectification phenomenon and the thermal rectification ratio increases with the increase in graphene/BN length. This phenomenon can be well explained by the mismatch of out-of-plane phonon vibration modes in two directions at the interface. Then, we explored the underlying mechanism of the length dependence of the thermal transport properties. For instance, the difference between the length dependence of the heat conduction in two directions reflects the change in the thermal rectification ratio. The yielded result shows the potential of graphene/BN heterostructure to act as an active thermal management element for semiconductor devices.

## Figures and Tables

**Table 1:** Comparison of our results with others in the forward direction.

Data	Ref [35]	Ref [36]	This Work
Thermal resistance (Km^2^/W)	2.6 × 10^−10^	/	2.2 × 10^−10^
Thermal conduction (MW/mK)	/	752.7 *	1138.5
Thermal conduction (MW/m^2^K)	/	/	4554

* Value at system length of 60nm. This does not affect the order of magnitude comparison.

## Data Availability

The data presented in this study are available in the article.
